# The fiendish behavior of TNF can be counteracted by microRNA

**DOI:** 10.15252/emmm.201505380

**Published:** 2015-06-13

**Authors:** Jean-Marc Cavaillon

**Affiliations:** Unit Cytokines & Inflammation, Institut PasteurParis, France

## Abstract

Tumor necrosis factor (TNF) is a fascinating anti-tumoral cytokine, which plays a key role in orchestrating the fight of innate immunity against infection. Concomitantly, TNF is a major player of the inflammatory response, as illustrated by successful therapeutic strategies targeting TNF in patho logies such as Crohn’s diseases, rheumatoid arthritis or psoriasis. In mice, TNF is able to induce tissue injuries and lethal shock. In this issue of *EMBO Molecular Medicine,* Puimège *et al* (2015) elegantly demonstrated that the lethal shock induced by TNF reflects high levels of its receptor TNFRI as seen in sensitive (*Mus musculus*), vs. resistant (*Mus spretus*) mice, where TNFR1 expression is low. They reported that this expression is under the control of a microRNA (miR-511), which itself is induced by glucocorticoids.

See also: **L Puimège *et al*** (August 2015)

In 1898, William Coley demonstrated that bacterial products have the capacity to allow the regression of tumors in patients. In 1975, Carswell *et al* discovered that injection of BCG and endotoxin of Gram-negative bacteria (lipopolysaccharide, LPS) in mice led to the production of “tumor necrosis factor” (TNF), a cytokine with tumor-necrotizing properties. Although TNF was discovered for its anti-tumoral activity, its use in cancer has been rather limited to rare cases of soft tissue sarcoma in limbs that allow isolated limb perfusion (Bonvalot *et al*, 2009). This limited clinical application is due to its high toxicity. Similar to many cytokines, TNF has yin and yang properties. As a main director orchestrating innate immunity, TNF is a prerequisite to fight bacterial infection and also contributes to address fungal, parasitic and viral infections. But, concomitantly, TNF is a key cytokine in inflammation, responsible for the deleterious events associated with many inflammatory disorders. For instance, TNF-targeting treatments in Crohn’s disease, rheumatoid arthritis or psoriasis, which lead to significant improvements, illustrate the dark side of TNF. Nevertheless, the collateral damages of these treatments, namely increased occurrence of infections (tuberculosis, sepsis…) and increased rate of lymphoma, further illustrate the major contribution of TNF to control the occurrence of these events.

TNF was the first cytokine to be detected in the bloodstream of patients with sepsis (Waage *et al*, 1986). This discovery, later followed by the finding that all pro- and anti-inflammatory cytokines could be detected in the plasma of sepsis patients, illustrates the so-called “cytokine storm”, well known to be associated with organ failure as seen in patients with sepsis, and even in non-infectious settings (Suntharalingam *et al*, 2006). Indeed, for many cytokines, a correlation exists between high plasma levels or persistence of detectable levels and poor outcome. Injection of TNF in mice can result in kidney, lung and small bowel injuries, and eventually death if high concentrations are injected (Tracey *et al*, 1986). Furthermore, in animal models, approaches to neutralize TNF have been successful to protect against LPS-induced shock or sepsis.

Mainly produced by pathogen-associated molecular patterns (PAMPs) or damage-associated molecular patterns (DAMPs) activated monocytes/macrophages, TNF is a homotrimer molecule released from the cell surface by a specific enzyme (TNFα-converting enzyme, TACE). It acts through two different TNF receptors (TNFR1 or p55, and TNFR2 or p75). The binding of TNF to the extracellular domains of TNFR1 bridges the receptors, thus gathering the death domains of the cytoplasmic moiety and initiating the signaling cascade ending either in apoptosis or in the production of many inflammatory mediators (eicosanoids, proteases, free radicals, adhesion molecules…). TNF also induces the release of other cytokines, which can act in synergy with TNF within an amplificatory feedback loop (e.g. interferon-γ (IFNγ) and interleukin-1β (IL-1β)), or which drive TNF-induced lethal shock (i.e. type I interferon, Huys *et al*, 2009). In TNFR1 KO mice, both the negative impact of the deletion of the receptor in *Listeria monocytogenes* infection, and the beneficial consequence when mice were injected with endotoxin or a Gram-positive-derived exotoxin were demonstrated (Pfeffer *et al*, 1993). However, mice exhibit high resistance to endotoxin or exotoxin toxicity, making the process difficult to study *in vivo*. To tackle this drawback, a “dirty little secret” is employed, namely D-galactosamine, a hepatotoxic agent, which is injected together with the bacterial toxin (NB D-galactosamine was also used when bacterial DNA was shown to induce lethal shock in mice). In this experimental setting, the process becomes fully TNF dependent. Noteworthy, the LD50 of LPS was similar in wild-type and TNF KO mice, whereas, as compared to wild-type mice, TNF-deficient mice were far more resistant when LPS was administrated together with D-galactosamine (Amiot *et al*, 1997a,b). These results illustrate the major role of the liver in the deleterious effects of TNF.

While most investigations are carried out in the laboratory mice belonging to the *Mus musculus* species, there are other species closer to wild-type animals, such as *Mus spretus* that display some specific properties, particularly in terms of resistance to bacterial and viral infection. Of interest is the possibility to cross these mice despite an estimated 1.5 million years of evolutionary divergence between them. The SPRET/Ei *M. spretus* are highly resistant to LPS-induced lethal shock, and the same team showed that this property was linked to a defective interferon-β production (Mahieu *et al*, 2006). Furthermore, SPRET/Ei *Mus spretus* are also highly resistant to the lethal effects of TNF while C57BL/6 *Mus musculus* are highly sensitive.

**Figure 1:**
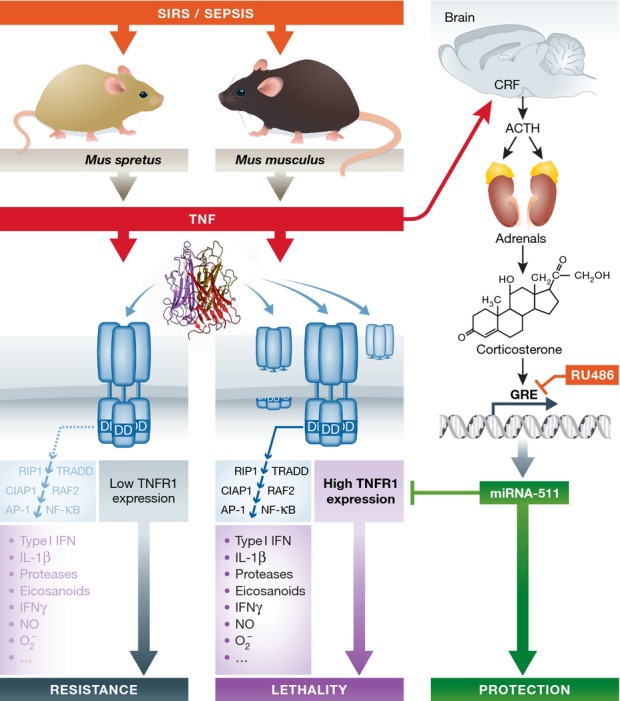
The expression of TNF receptor type I is under the control of miRNA The toxic properties of TNF in laboratory mice (*Mus musculus*) and not in wild-type mice (*Mus spretus*) are linked to the level of TNF receptor I (TNFR1) expression in all tissues of the respective rodents. The binding of the homotrimeric TNF molecule to its receptor favors contacts between the death domains (DD) of the cytoplasmic moieties of the TNFRI. The receptor activation initiates an intracellular signaling cascade that leads to the production of numerous inflammatory mediators, which contribute to the deleterious effects of TNF. TNFR1 expression is under the control of a microRNA (miR-511), of which the production is regulated by glucocorticoids. Glucocorticoids themselves can be induced by TNF following its activation of the hypothalamic–pituitary–adrenal axis, leading to the sequential release of corticotropin-releasing factor (CRF) and adrenocorticotrophic hormone (ACTH). Different levels of corticosterone and miR-511 in *M. spretus* and *M. musculus* correlate with their respective sensitivity to TNF. A glucocorticoid receptor antagonist (RU486) can obliterate the resistance of *Mus spretus* to TNF.

In their recent work, Puimège *et al* (2015) deciphered the reasons of the difference of the respective sensitivity of these mice when exposed to TNF. Claude Libert’s group most elegantly demonstrated with genetic, immunological and molecular biological approaches that resistance was reflecting a low expression of TNFRI in *M. spretus* in all studied tissues (liver, kidney, lung, spleen) independently of any natural shedding; indeed, soluble TNFRI levels in plasma were also lower in *M. spretus* as compared to that in *M. musculus*. They established that the 10 amino acid difference of TNFRI in both mouse strains was not associated with any functional changes. Since similar levels of TNFRI mRNA were found in both types of mice, they focused on TNFRI translational regulation. They discovered that a microRNA (miR-511) could down-regulate TNFRI translation, and observed a higher expression of miR-511 in *M. spretus* mice. The most fascinating were the experiments in which the authors were able to protect sensitive C57BL/6 mice against TNF-induced lethality with hydrodynamic injection of plasmids expressing miR-511 and to sensitize resistant SPRET/Ei mice with plasmids expressing anti-miR-511. These approaches are said by the authors to only target the liver. To further complete the beauty of their demonstration, using glucocorticoid receptor antagonist (RU486), adrenalectomized mice and dexamethasone and measuring circulating corticosterone, they convincingly established that miR-511 is under the control of glucocorticoids.

This work enlightens the complexity of the regulatory loops involved in inflammation. TNF can activate the hypothalamic–pituitary–adrenal axis that results in the release of glucocorticoids, cytoplasmic receptors being themselves regulated by miRNAs. Once complexed to their receptors, glucocorticoids bind to glucocorticoid response elements (GRE), present in the promoters of numerous genes, favoring or inhibiting the transcription. Anti-inflammatory properties of glucocorticoids reflect their capacity to regulate the transcription of more than 900 GRE-element-containing genes, supporting the production of annexin-A1 and IL-10, and inducing the release of certain miRNAs. The demonstration of the induction of miR-511 by glucocorticoids and its capacity to limit the expression of TNFRI is a new clue that allows a better understanding of the complex control of inflammation by glucocorticoids.
